# The Role of Saffron in the Treatment of Depression: A Literature Review

**DOI:** 10.7759/cureus.104594

**Published:** 2026-03-03

**Authors:** Luigi Dimech

**Affiliations:** 1 Medicine, Mater Dei Hospital, Msida, MLT

**Keywords:** adjunctive therapy, major depressive disorder (mdd), nutraceuticals, randomised controlled trials, saffron

## Abstract

Saffron (*Crocus sativus* L.) has attracted increasing interest as a nutraceutical option for depressive disorders, particularly for patients who experience incomplete response or poor tolerability with conventional treatments. Importantly, clinical studies have evaluated extracts derived from different plant parts, most commonly the stigma (the culinary “saffron” spice) and, in some trials, the petal, which have distinct constituent profiles and therefore differ in interpretability and generalisability.

This literature review synthesises evidence from randomised controlled trials, meta-analyses, safety data, dosing patterns, proposed mechanisms, and guideline positioning regarding saffron in depression, with a focus on adult major depressive disorder and related depressive symptomatology. Across placebo-controlled trials in mild-to-moderate depression, saffron, most commonly administered at 30 mg per day for approximately six weeks, has been associated with clinically meaningful reductions in depressive symptom severity, typically measured using the Hamilton Depression Rating Scale (HAM-D).

Small head-to-head studies suggest broadly comparable short-term symptom improvement between saffron and standard antidepressants such as fluoxetine and imipramine, although these trials are generally underpowered and not designed to establish formal non-inferiority. Adjunctive studies, including trials of standardised extracts (e.g., Affron®) and constituent-focused interventions (notably crocin), provide preliminary support for augmentation in partial responders, but findings are heterogeneous and long-term outcomes remain unclear. Meta-analytic evidence generally indicates superiority over placebo and similar efficacy to selective serotonin reuptake inhibitors (SSRIs), with an overall favourable short-term tolerability profile; however, confidence is constrained by small sample sizes, restricted settings, variable product standardisation (including stigma versus petal preparations), and risk of bias/publication bias. Saffron’s putative antidepressant effects may involve modulation of monoaminergic pathways alongside antioxidant, anti-inflammatory, and neuroprotective actions. Current guidelines position saffron as a third-line complementary option for mild depressive episodes and as a potential adjunct in moderate depression.

Overall, saffron appears promising for short-term symptom reduction in mild-to-moderate depression, but larger, longer, multi-centre trials using standardised preparations and clinically meaningful endpoints are required to clarify durability, comparative effectiveness, safety, and optimal clinical use.

## Introduction and background

Depression is a leading cause of disability worldwide and is characterised by sustained low mood, anhedonia, anergia, and a range of associated cognitive and somatic symptoms [1]. Although standard first-line approaches, such as lifestyle interventions, psychotherapy, and antidepressant medication, are effective for many patients, a meaningful minority experience partial response, intolerable adverse effects, or poor adherence [2]. These limitations have contributed to growing interest in adjunctive and alternative strategies, including the use of nutraceuticals, which are food-derived products used for potential health benefits beyond basic nutrition.

Often nicknamed “red gold,” saffron is widely regarded as one of the most expensive culinary spices. It is obtained from the dried stigmas of *Crocus sativus *L. [3]. Beyond its role as a food additive, saffron has a long history as a therapeutic plant. Traditionally, it has been used to ease gastrointestinal discomfort and spasms, support digestion, stimulate appetite, and act as an antihypertensive [4,5]. Preclinical studies also suggest potential anticancer effects, with saffron and its constituents reported to inhibit tumour formation and/or slow tumour progression across a range of experimental animal models [4,6].

In Persian traditional medicine, saffron has also been used to treat depressive symptoms [7]. Interest in saffron’s psychotropic potential has focused on several bioactive constituents, particularly the carotenoids crocin and crocetin, and the volatile compound safranal [8]. Clinical studies have primarily investigated saffron either as monotherapy for mild-to-moderate depressive disorders or as an adjunct to conventional antidepressant treatment. This literature review summarises key randomised controlled trials (RCTs), meta-analyses, safety data, dosing patterns, and guideline positioning for saffron in depressive disorders, with emphasis on adult major depressive disorder (MDD) and related depressive symptomatology.

## Review

Methods

Databases and search strategy: A targeted search was undertaken in PubMed (primary database), with supplementary searching via Google Scholar and reference-list screening of key trials and evidence syntheses included in the bibliography. Searches covered database inception to May 2025, with inclusion of major evidence syntheses published in 2025. Search terms combined keywords relating to the intervention and condition, including: 'saffron', '*Crocus sativus'*, 'crocin', 'crocetin', 'safranal', 'depression', and 'meta-analysis'.

Eligibility Criteria

Included studies were peer-reviewed human studies in adults (≥18 years) with MDD or clinically significant depressive symptoms that evaluated saffron as stigma-derived extracts, petal-derived preparations, or isolated constituents, as monotherapy or adjunctive/augmentation treatment. Priority was given to randomised controlled trials, systematic reviews/meta-analyses, and relevant clinical guidelines. Excluded were non-human studies (except where briefly cited for mechanistic context), case reports/series, conference abstracts without sufficient methodological detail, and studies without depressive outcomes.

Study Selection and Synthesis

Titles/abstracts were screened for relevance, and full texts were reviewed where appropriate. Reference lists of included RCTs and meta-analyses were hand-searched to identify additional eligible studies. Evidence was synthesised thematically (placebo-controlled trials; head-to-head comparisons versus antidepressants; stigma versus petal preparations; adjunctive trials; meta-analytic evidence; safety/tolerability; mechanisms; guideline positioning), with interpretation explicitly considering heterogeneity in saffron source and product standardisation.

Risk of Bias Assessment

A formal risk-of-bias assessment tool was not applied, consistent with the narrative design; however, study limitations (e.g., sample size, duration, setting, and product heterogeneity) and potential publication bias highlighted by meta-analyses were considered when interpreting findings.

Evidence from clinical trials

Clinical trials in participants with mild-to-moderate depressive disorders have most commonly evaluated saffron at a total daily dose of 30 mg, typically administered in divided doses of 15 mg twice daily (morning and evening). Follow-up has usually been around six weeks, with symptom severity most often assessed using the Hamilton Depression Rating Scale (HAM-D) [9,10].

Saffron as Monotherapy versus Placebo

Results from placebo-controlled RCTs have consistently revealed a marked reduction in depressive symptom scores with saffron compared to placebo. A similarly structured trial also revealed temporary improvement in symptoms without major tolerability concerns [9].

Saffron versus Standard Antidepressants

Data collected from several small head-to-head trials comparing the psychotropic effects of saffron and standard antidepressants have reproduced similar results. When compared to tricyclic antidepressants such as imipramine (100mg/day), saffron demonstrated comparable efficacy. It was also noted that anticholinergic and sedative effects occurred more frequently with imipramine [10]. Similarly, a small double-blind RCT comparing saffron to a selective serotonin reuptake inhibitor (SSRI) such as fluoxetine (20mg/day) revealed broadly similar improvements across groups [7]. This was reproduced in additional studies, which used specific clinical contexts, such as post-percutaneous coronary intervention and postpartum depression, with broadly similar adverse event rates [11,12].

These comparative trials are clinically noteworthy because symptom improvement with saffron appears broadly similar to that seen with standard antidepressants. However, most studies were small, of short duration, and were neither designed nor sufficiently powered to demonstrate formal non-inferiority. Similarly, in the above clinical contexts mentioned, whilst these studies broaden the population base, they, however, remain relatively small and short, and do not establish long-term relapse prevention or functional outcomes.

Alternative Saffron Source - Petal (Non-stigma) Preparations

Some RCTs have investigated petal preparations of *Crocus sativus* rather than the stigma. These plant parts are chemically distinct, and therefore clinical findings from petal-based products cannot be assumed to apply to stigma-derived extracts [8].

Two trials have evaluated petal-derived saffron at a total daily dose of 30 mg. In a six-week placebo-controlled study, participants receiving petal extract showed a greater reduction in depressive symptom severity, with consistently larger improvements in HAM-D scores than placebo [13]. In a separate eight-week trial comparing petal extract with fluoxetine 20 mg/day, petal-derived saffron demonstrated similar efficacy, with comparable reductions in depressive symptoms across the treatment period [14].

Overall, petal preparations appear to confer antidepressant benefits in the short term. However, because petals and stigmas differ in constituent profiles, these results should not be extrapolated to stigma-based commercial saffron extracts without caution. Further external replication and improved product standardisation are needed before petal-derived preparations can be confidently translated into routine clinical practice.

Adjunctive use of saffron (as an add-on to antidepressants)

A notable minority of patients treated with first-line conventional antidepressants experience only partial symptom amelioration or discontinue treatment due to undesirable side effects or poor tolerability [2]. This has prompted interest in adjunctive strategies that may enhance antidepressant response, improve tolerability, and support adherence. Trials have investigated saffron as an add-on intervention, either as a whole extract (including standardised preparations) or via its key bioactive constituents.

One randomised, double-blind, placebo-controlled study used a standardised saffron extract called Affron® as augmentation in adults with persistent depressive symptoms despite ongoing antidepressant therapy [15]. Adjunctive saffron was associated with greater improvement in clinician-rated depressive symptoms compared with placebo over the treatment period, supporting a potential role for saffron in partial responders. However, effects were not uniformly demonstrated, with some self-rated outcomes failing to clearly differentiate from placebo. Interpretation is further limited by sample size, duration, and heterogeneity (e.g, different saffron sources, dosing, depression severity) in concurrent antidepressant regimens [15].

Combination strategies have also been examined. In a 12-week randomised, double-blind, placebo-controlled trial of 123 adults with major depressive disorder, participants received placebo, low-dose curcumin (250 mg twice daily), high-dose curcumin (500 mg twice daily), or low-dose curcumin plus saffron (15 mg twice daily) [16]. Pooled active treatments produced greater improvements in self-rated scales for depressive and anxiety symptoms than placebo, with stronger responses in atypical depression. However, there were no significant differences between curcumin doses or between curcumin alone and the curcumin-saffron combination, suggesting no clear incremental benefit from adding saffron in this study [16].

Adjunctive effects have also been examined using isolated saffron constituents, particularly crocin. A small double-blind pilot RCT reported that crocin, added to SSRIs in major depressive disorder, was associated with greater improvement in depressive symptomatology compared with a placebo add-on. While this supports biological plausibility for saffron-derived augmentation, the evidence base remains early-stage [17].

All in all, these adjunctive findings are encouraging; the evidence remains preliminary. The available trials are limited by their small sample size, short follow-up, and limited data on remission, functional recovery, and relapse prevention, and therefore require replication in larger, longer, methodologically robust trials.

Evidence synthesis

A widely cited 2019 systematic review and meta-analysis pooling 23 studies reported a significant reduction in depressive symptoms with saffron compared with placebo (Hedges’ g ≈ 0.99), and a similar massive effect when saffron was used as an adjunct to antidepressants (g ≈ 1.23) [18]. Subsequent head-to-head meta-analytic evidence in April 2023 found no significant difference in symptom reduction between saffron and SSRIs, while suggesting fewer adverse events with saffron [19]. More recently, a 2025 network meta-analysis of nutraceuticals (192 trials; >17,000 participants; 44 agents) identified several nutraceuticals, including saffron, with potential benefit as monotherapy or adjunctive therapy [20]. As this review does not perform original statistical synthesis, effect sizes are presented as reported in the included meta-analyses; where 95% confidence intervals and/or p-values were provided by the source papers, these were prioritised in interpretation, alongside their discussion of heterogeneity, risk of bias, and potential publication bias. However, confidence in these estimates remains limited by small trial sizes, restricted settings and populations, and heterogeneity in saffron preparations and study methodologies.

Safety and tolerability

As stated previously, most depression trials have used saffron at a total daily dose of 30 mg [18]. Across these short-term RCTs, saffron appears generally well-tolerated, with adverse events typically denoted to be mild and not significantly different from comparator groups [18]. Reported side effects tend to be limited to gastrointestinal upset, dizziness, headache, and occasional mild sedation.

Broader toxicology literature in both animal and human studies suggests low acute toxicity at typical exposures, but indicates potential harm at substantially higher doses, such as over 5 grams, and highlights the relative paucity of robust long-term safety data in humans [21]. As with other nutraceuticals, clinical use is complicated by variability in supplement quality and standardisation, the possibility of adulteration, and the potential for interactions, particularly in patients prescribed multiple psychotropics. Caution is also advised in pregnancy and breastfeeding, given limited high-quality safety evidence at supplemental doses [21].

Notably, one randomised trial reported that saffron may improve SSRI-associated sexual dysfunction (examined in women taking fluoxetine) [22]. If confirmed in further studies, this effect could have pragmatic value by improving tolerability and potentially supporting adherence in selected patients.

Proposed mechanisms of action of saffron

Saffron’s putative antidepressant effects are postulated to arise from a combination of neurochemical modulation and neurobiological protective mechanisms, with crocin/crocetin and safranal most frequently implicated. Preclinical evidence suggests saffron may enhance mood-related neurotransmission by influencing monoaminergic pathways (including serotonin, dopamine, and noradrenaline), with some data indicating reuptake inhibition mechanisms analogous to conventional antidepressants [8].

In parallel, saffron demonstrates antioxidant, anti-inflammatory, and neuroprotective properties, including free-radical scavenging, downregulation of pro-inflammatory cytokines, and mitigation of neuronal apoptosis. Additional proposed actions include modulation of glutamatergic signalling, inhibition of acetylcholinesterase, regulation of stress systems such as the hypothalamic-pituitary-adrenal (HPA) axis, and promotion of neuroplasticity through activation of brain-derived neurotrophic factor (BDNF) pathways; effects on gene expression related to cellular repair and stress responses have also been described [8].

Current guidelines

The Canadian Network for Mood and Anxiety Treatments (CANMAT) is an expert-led collaboration focused on improving the assessment and treatment of mood and anxiety disorders. In its guideline update published in The Canadian Journal of Psychiatry (2024), CANMAT discusses *Crocus sativus *L. within the section on complementary and alternative medicine (CAM) for depressive disorders. It classifies saffron as a third-line CAM option for mild-severity major depressive episodes, citing the modest overall evidence (level 2 evidence) and important limitations within the available randomised controlled trial base [2].

CANMAT further emphasises that CAM interventions, including saffron, do not have an evidence base comparable to first-line psychotherapy or antidepressant pharmacotherapy for moderate-to-severe depression. Accordingly, it suggests that CAM treatments should be considered as monotherapy only in mild depression, with use as an adjunct alongside standard treatments in moderate severity illness [2].

## Conclusions

Overall, saffron illustrates promising short-term psychotropic effects in mild-to-moderate depression, with small RCTs and meta-analyses reporting efficacy superior to placebo and often broadly comparable to antidepressants such as SSRIs and TCAs at commonly studied doses (typically 30 mg/day over six weeks). Adjunctive trials, including standardised extracts and constituent-focused studies (e.g., crocin), suggest potential utility in selected patients, particularly where tolerability and patient preference are important considerations. However, the evidence base remains limited by small sample sizes, short follow-up, and heterogeneity of preparations (including stigma vs petal products and variable standardisation), restricting confidence in long-term effectiveness, safety, and generalisability. These limitations underpin guideline caution, reflected in CANMAT’s third-line positioning of saffron as a complementary option for mild depressive episodes rather than a first-line treatment. 

Accordingly, future research should prioritise adequately powered, multi-centre trials using chemically characterised and standardised preparations with explicit reporting of constituent content and saffron source. Pragmatic non-inferiority RCTs comparing stigma-derived saffron with first-line SSRIs (e.g., escitalopram), with at least 12-month follow-up, would be particularly informative, incorporating endpoints beyond symptom reduction, such as remission, time to relapse/recurrence, functional recovery (work and social functioning), quality of life, sexual functioning/tolerability, and adherence. In parallel, adjunctive RCTs in partial responders on stable antidepressant regimens, stratified by relevant clinical features and powered for remission and functional outcomes, are needed to clarify the durability of benefit, optimal formulations, comparative effectiveness, and safety across broader populations.
